# Standardizing the classification of gastric cancer patients with limited and adequate number of retrieved lymph nodes: an externally validated approach using real-world data

**DOI:** 10.1186/s40779-022-00375-2

**Published:** 2022-04-07

**Authors:** Wei Wang, Yu-Jie Yang, Ri-Hong Zhang, Jing-Yu Deng, Zhe Sun, Sharvesh Raj Seeruttun, Zhen-Ning Wang, Hui-Mian Xu, Han Liang, Zhi-Wei Zhou

**Affiliations:** 1 Department of Gastric Surgery, Sun Yat-Sen University Cancer Center, Guangzhou, 510060 China; 2 State Key Laboratory of Oncology in South China, Collaborative Innovation Center for Cancer Medicine, Guangzhou, 510060 China; 3 Department of Gastric Cancer Surgery, Tianjin Medical University Cancer Institute and Hospital, Tianjin, 300000 China; 4 Department of Surgical Oncology, the First Hospital of China Medical University, Shenyang, 110000 China

**Keywords:** Lymph nodes, Limited, Adequate, Gastric cancer, American Joint Committee on Cancer, Tumor-node-metastasis, Staging system, Overall survival

## Abstract

**Background:**

Currently, there is no formal consensus regarding a standard classification for gastric cancer (GC) patients with < 16 retrieved lymph nodes (rLNs). Here, this study aimed to validate a practical lymph node (LN) staging strategy to homogenize the nodal classification of GC cohorts comprising of both < 16 (Limited set) and ≥ 16 (Adequate set) rLNs.

**Methods:**

All patients in this study underwent R0 gastrectomy. The overall survival (OS) difference between the Limited and Adequate set from a large Chinese multicenter dataset was analyzed. Using the 8th American Joint Committee on Cancer (AJCC) pathological nodal classification (pN) for GC as base, a modified nodal classification (N’) resembling similar analogy as the 8th AJCC pN classification was developed. The performance of the proposed and 8th AJCC GC subgroups was compared and validated using the Surveillance, Epidemiology, and End Results (SEER) dataset comprising of 10,208 multi-ethnic GC cases.

**Results:**

Significant difference in OS between the Limited and Adequate set (corresponding N0–N3a) using the 8th AJCC system was observed but the OS of N0_limited_ vs. N1_adequate_, N1_limited_ vs. N2_adequate_, N2_limited_ vs. N3a_adequate_, and N3a_limited_ vs. N3b_adequate_ subgroups was almost similar in the Chinese dataset. Therefore, we formulated an N’ classification whereby only the nodal subgroups of the Limited set, except for pT1N0M0 cases as they underwent less extensive surgeries (D1 or D1 + gastrectomy), were re-classified to one higher nodal subgroup, while those of the Adequate set remained unchanged (N’0 = N0_adequate_ + pT1N0M0_limited_, N’1 = N1_adequate_ + N0_limited (excluding pT1N0M0limited)_, N’2 = N2_adequate_ + N1_limited_, N’3a = N3a_adequate_ + N2_limited_, and N’3b = N3b_adequate_ + N3a_limited_). This N’ classification demonstrated less heterogeneity in OS between the Limited and Adequate subgroups. Further analyses demonstrated superior statistical performance of the pTN’M system over the 8th AJCC edition and was successfully validated using the SEER dataset.

**Conclusion:**

The proposed nodal staging strategy was successfully validated in large multi-ethnic GC datasets and represents a practical approach for homogenizing the classification of GC cohorts comprising of patients with < 16 and ≥ 16 rLNs.

**Supplementary Information:**

The online version contains supplementary material available at 10.1186/s40779-022-00375-2.

## Introduction

Gastric cancer (GC) is the third leading cause of cancer-related death worldwide [1, 2] and has a low 5-year relative survival of 32.4% [3]. Early lymph node (LN) metastasis is among the prime factors responsible for this dismal prognosis and its extent guides post-operative management. Therefore, proper LN classification is important for accurate survival prognostication as this would lead to better treatment selection and improved therapeutic outcomes.

The American Joint Commission on Cancer (AJCC) staging manual [4], National Comprehensive Cancer Network (NCCN) GC guidelines [5], and the Chinese Society of Clinical Oncology (CSCO) guidelines [6] for the diagnosis and treatment of GC recommend the retrieval of at least 15 LNs for proper staging of resectable GC. However, there have been some ongoing and unresolved debates regarding perigastric lymphadenectomies. First, one study, which was among the key references for the development of the 8th AJCC GC staging system, proposed a new stage grouping based on the data of 25,411 GC patients [mean and median number of retrieved lymph nodes (rLNs), 34.9 and 32.0, respectively] collected from 59 institutions globally [7]. Although its data could be accurately reflecting the lymphadenectomies and gastrectomies performed at high-volume institutions (high expertise for retrieving > 15 LNs), however, performing such high-quality surgeries are particularly challenging in the daily practice of lower-volume institutions (lesser expertise and higher risk of retrieving < 15 LNs) [8–10]; especially considering that the number of surgeries performed at these lower-volume institutions could agglomerate to a large proportion of GC surgeries performed globally. So, whether the 8th AJCC GC staging system is as optimal for institutions having considerable proportion of advanced GC cases with sub-optimal rLNs remains to be determined.

Second, studies have found that the survival of patients with < 15 rLNs is worse than those with > 15 rLNs [5-year overall survival (OS) of corresponding N0 to N3 of < 15 rLNs vs. > 15 rLNs cohorts: 76.4–19.2% vs. 87.5–24.8%, respectively] [11–14]. It has been further reported that cases with < 10–15 rLNs were more likely to be under-staged [15] and the 5-year survival of patients with ≤ 6 rLNs would significantly improve for every 10 extra rLNs [16]. Considering that in real-world practice almost all GC lymphadenectomy-performing hospitals have cases where < 15 LNs were surgically retrieved, one of the main differences between hospitals is the ratio of limited and adequately rLNs cases. Despite understanding the survival differences between these two sets of patients, there are no standard criteria on how to merge these patients within the tumor-node-metastasis (TNM) subgroups in hospitals GC datasets. Clinically, both sets of patients are still classified within the same TNM subgroups and these datasets are used as reference, which can be misleading, when assessing new individual patient’s prognosis for treatment planification, and could therefore lead to inaccuracies in survival estimation and possible mismanagement of subgroups of GC patients.

Thus, the aims of this study were to formulate and validate a modified nodal classification strategy to homogeneously classify GC patients with < 15 and ≥ 15 rLNs using a staging analogy as close as possible to that of the 8th AJCC N stage for easy practical applicability but with lesser survival heterogeneity between corresponding subgroups of patients within these two nodal categories.

## Methods

### Patients and eligibility criteria

A multicenter retrospective study. The data from a large Chinese multicenter dataset consisting of 10,526 patients who underwent gastrectomies at the Sun Yat-sen University Cancer Center (SYSUCC; Guangzhou, China), the First Hospital of China Medical University (CMU; Shenyang, China), and Tianjin Medical University Cancer Institute & Hospital (TJMU; Tianjin, China) from January 1, 2000, to December 31, 2012, were analyzed. Patients satisfying the following criteria were included: pathologically confirmed primary gastric adenocarcinoma; no other synchronous malignancy; absence of residual GC; no preoperative chemotherapy and distant metastasis prior to surgery; gastrectomies and lymphadenectomies (limited or extended) performed according to the Japanese Gastric Cancer Treatment Guidelines 2014, Version 3; R0 resection (no residual macroscopic or microscopic tumor); postoperative survival of at least 3 months; and no missing data for the proper TNM evaluation of the patients. A flow diagram of the selection process is presented in Additional file 1: Fig. S1. All patients provided informed consent for the use of their data for scientific purposes prior to surgery. The study protocol adhered to the regulations of the Declaration of Helsinki (as revised in Edinburgh 2000) and ethical approval was obtained from the Sun Yat-Sen University Cancer Center institutional review board (B2022-161-01).

### Retrieval of patients from the SEER database

We examined the SEER database for GC cases conforming to similar inclusion criteria [i.e., R0 gastrectomy and lymphadenectomies (limited or extended), etc.] mentioned above from January 1998 to December 2012. Initially, 31,988 cases from 18 registries were retrieved. Those with incomplete information regarding age, tumor size, tumor location, Lauren type, depth of tumor invasion, LNs, non-radical resection, and status of distant metastasis were excluded. This dataset was defined as the SEER validation dataset.

#### Patients’ stratification and study endpoints

In the Chinese and SEER datasets, patients with < 16 and ≥ 16 pathologically rLNs were stratified as two categories, and defined as the Limited and Adequate sets, respectively. They were then restaged according to the 8th AJCC staging system. The study endpoint was OS, defined as the time interval from the date of surgery to the last date of follow-up or death. We also aimed to find an easy but practical approach, using a staging analogy as close as possible to that of the 8th AJCC N stage, to classify patients from both sets within corresponding nodal subgroups where the OS of the Limited set would approximate that of the Adequate set.

### LN analysis

The statistical differences in OS of each pathological nodal subgroups between these two sets of patients were analyzed and based upon the differences observed, a modified nodal classification scheme (N’) was proposed to reclassify the patients from the Limited set so that within each N’ subgroups, the OS of the Limited set approximated that of the Adequate set. For overall stage classification assessment, the pN of the 8th AJCC pTNM was then replaced by our proposed N’ classification and termed as the pTN’M classification.

The prognostic performances of these two nodal classifications and staging systems were assessed in terms of discriminatory ability, i.e., differences in OS among patients in different stages (log-rank *χ*^2^ test); monotonicity, i.e., association between stages and OS (linear-trend *χ*^2^ test); and homogeneity, i.e., differences in OS among patients within the same stage (likelihood-ratio *χ*^2^ test). The Akaike information criterion (AIC) was used to measure the optimal prognostic stratification of both sets of patients. Higher log-rank *χ*^2^ and linear-trend *χ*^2^ scores indicated better discriminatory ability and monotonicity, higher likelihood-ratio *χ*^2^ score indicated greater homogeneity. Smaller AIC values represented more optimal prognostic stratification.

### Statistical analysis

The Kaplan–Meier method was used for graphical representation of the OS curves and the log-rank test was used to assess the differences between the survival curves. To identify risk factors associated with each nodal classification and staging system, univariate and multivariate analyses were performed using the log-rank test. The log-rank *χ*^2^ test, linear-trend *χ*^2^ test, likelihood-ratio *χ*^2^ test, and the AIC within the Cox regression model were utilized to compare the performance of the two staging systems. Hazard ratios (*HR*) and 95% confidence intervals (95%CI) were also generated. Statistical analyses were performed using the Statistical Package for the Social Sciences (SPSS) software (version 21.0, SPSS Inc., Chicago, IL) and R statistical software (version 3.3.1, the R Foundation for Statistical Computing). A *P* value < 0.05 (2-sided) was considered as statistically significant.

## Results

### Clinicopathological features of the Chinese and SEER datasets

The patients' clinicopathological features of both datasets are listed in Additional file 2: Table S1. Of the 7911 patients from the Chinese dataset, 89.9% of them had advanced GC. A total of 182,215 LNs were retrieved (range 1–118), with an overall number of (23 ± 13) rLNs per patient. For the Limited (*n* = 2414, 30.5%) and Adequate (*n* = 5497, 69.5%) sets, the number of LNs retrieved were (10 ± 4) and (28 ± 12), respectively. Of the 10,208 SEER cases analyzed, 83.2% had advanced disease and for a total number of 176,566 (range 1–90) LNs retrieved, the number of rLNs per patient for the Limited (*n* = 5429, 53.2%) and Adequate (*n* = 4779, 46.8%) sets were (9 ± 4) and (27 ± 12), respectively. In both datasets, univariate analyses showed that the parameters age, tumor location, tumor size, Lauren type, pT classification, pN classification, modified pN classification (N’ classification), the number of LNs retrieved, the 8th pTNM classification, and the modified pTNM classification (pTN’M classification) were correlated with prognosis, except for sex in the Chinese dataset (*P* = 0.082; Additional file 2: Table S1).

### Proposal of a modified nodal classification and staging system using the Chinese dataset

**Fig. 1:**
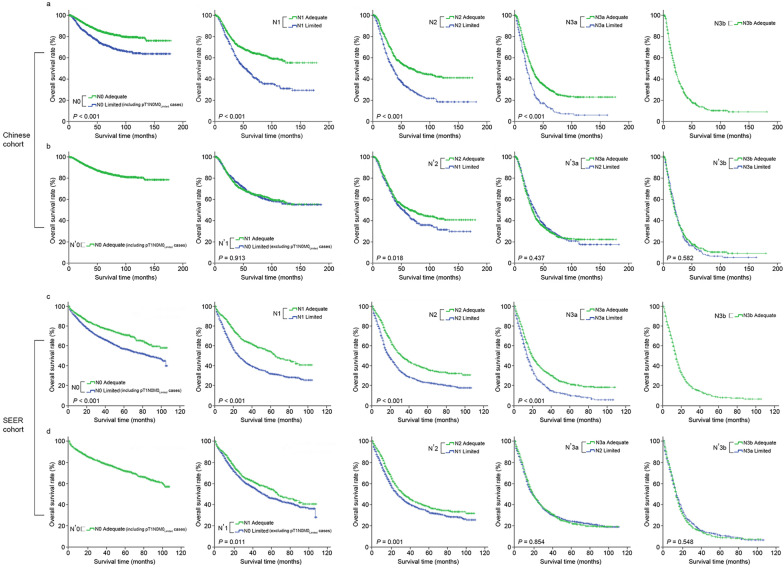
Graphical illustration showing the statistical differences between the overall survival of the different subgroups of pN (**a**, **c**) and pN’ (**b**, **d**) for the Limited and Adequate sets of the Chinese (**a**, **b**) and SEER (**c**, **d**) datasets. pN pathological nodal classification, pN’ modified pathological nodal classification

**Fig. 2:**
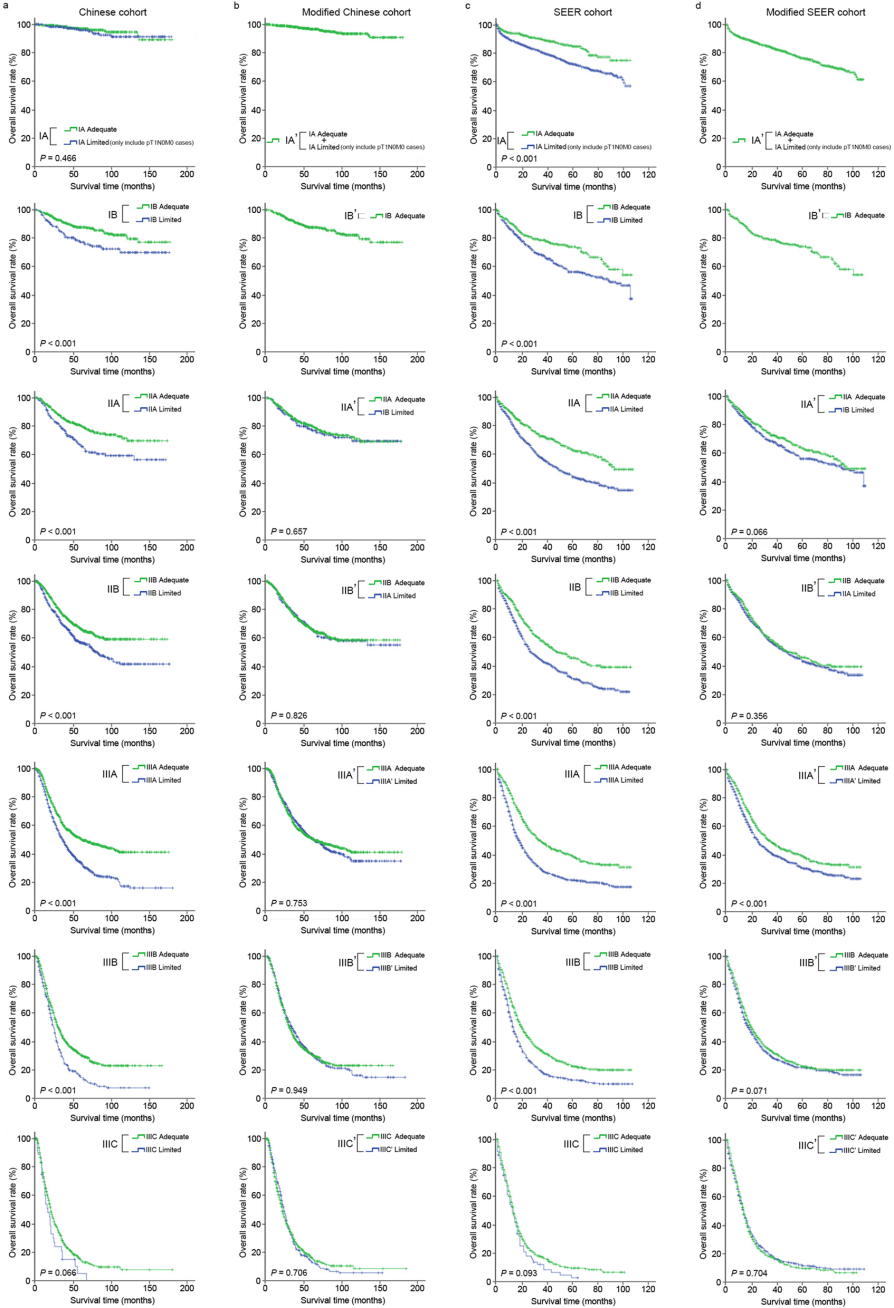
Graphical illustration showing the statistical differences between the overall survival of the different substages of pTNM (**a**, **c**) and pTN’M (**b**, **d**) for the Limited and Adequate sets of the Chinese (**a**, **b**) and SEER (**c**, **d**) datasets. pTNM pathological tumor-node-metastasis classification, pTN’M pathological tumor-modified node-metastasis classification

Kaplan Meier analysis was performed to obtain the statistical associations in OS between the 8th AJCC N subgroups of patients from the Limited and Adequate set. A significant difference in OS within the corresponding N0 to N3a subgroups between the Limited and Adequate sets was observed (*P* < 0.001; Fig. 1a). Using the 8th AJCC TNM classification, similar observations between the two sets were also observed (Limited vs. Adequate set), except for stage IA (*P* = 0.466) and stage IIIC (*P* = 0.066) (Fig. 2a).

Tables 1 and 2 show the detailed OS of the 8th AJCC pN stage. Upon stratifying it into its respective Limited and Adequate subgroups, we observed that the OS of N0_limited_ to N3a_limited_ was closer to that of N1_adequate_ to N3b_adequate_ rather than their corresponding N0_adequate_ to N3a_adequate_, respectively (*P* < 0.001).

**Table 1:** Analysis of the 5-year overall survival rates of patients using the 8th AJCC pN classification stratified into limited (< 16 rLNs) and adequate (≥ 16 rLNs) rLN set (Chinese multicenter dataset)

Stage	Limited rLN dataset		Stage	Adequate rLN dataset		*P* value (Limited vs. Adequate rLN dataset)	Stage	Combined rLN dataset	
	*n* (%)	5-year OS (%)		*n* (%)	5-year OS (%)			*n* (%)	5-year OS (%)
pN (*n* = 2414)			pN (*n* = 5497)				pN (*n* = 7911)		
N0	1063 (44.0)	72.2		1807 (32.9)	84.0	< 0.001		2870 (36.3)	79.6
N1	518 (21.5)	45.5		885 (16.1)	65.5	< 0.001		1403 (17.7)	57.6
N2	558 (23.1)	32.0		989 (18.0)	51.2	< 0.001		1547 (19.6)	44.0
N3a	275 (11.4)	13.2		1132 (20.6)	30.3	< 0.001		1407 (17.8)	26.9
N3b	–	–		684 (12.4)	14.9	–		684 (8.6)	14.9
^*^pN’ (*n* = 2164)			^*^pN’ (*n* = 5747)				pN’ (*n* = 7911)		
N’0	–	–		2057 (35.8)^*^	85.5	–		2057 (26.0)	85.5
N’1	813 (37.6)	65.3		885 (15.4)	65.5	0.913		1698 (21.5)	65.3
N’2	518 (23.9)	45.5		989 (17.2)	51.2	0.018		1507 (19.0)	49.1
N’3a	558 (25.8)	32.0		1132 (19.7)	30.3	0.437		1690 (21.4)	30.9
N’3b	275 (12.7)	13.2		684 (11.9)	14.9	0.582		959 (12.1)	14.4
pTNM (*n* = 2414)			pTNM (*n* = 5497)				pTNM (*n* = 7911)		
IA	250 (10.4)	95.9		551 (10.0)	96.9	0.466		801 (10.1)	96.6
IB	227 (9.4)	77.3		508 (9.2)	87.8	< 0.001		735 (9.3)	84.4
IIA	234 (9.7)	66.4		465 (8.5)	80.2	< 0.001		699 (8.8)	75.5
IIB	587 (24.3)	57.3		912 (16.6)	66.8	< 0.001		1499 (18.9)	63.0
IIIA	785 (32.5)	34.2		1291 (23.5)	51.1	< 0.001		2076 (26.2)	44.5
IIIB	294 (12.2)	14.4		1046 (19.0)	30.0	< 0.001		1340 (16.9)	26.4
IIIC	37 (1.5)	5.0		724 (13.2)	14.0	0.066		761 (9.6)	13.6
^*^pTN’M (*n* = 2164)			^*^pTN’M (*n* = 5747)				pTN’M (*n* = 7911)		
IA’	–	–		801 (13.9)^*^	96.6	–		801 (10.1)	96.6
IB’	–	–		508 (8.8)	87.8	–		508 (6.4)	87.8
IIA’	227 (10.5)	77.3		465 (8.1)	80.2	0.657		692 (8.7)	79.2
IIB’	234 (10.8)	66.4		912 (15.9)	66.8	0.826		1146 (14.5)	66.7
IIIA’	890 (41.1)	51.0		1291 (22.5)	51.1	0.753		2181 (27.6)	51.1
IIIB’	515 (23.8)	30.7		1046 (18.2)	30.0	0.949		1561 (19.7)	30.2
IIIC’	298 (13.8)	11.7		724 (12.6)	14.0	0.706		1022 (12.9)	13.3

^*^Contained patients with pT1N0M0 lesions (Chinese dataset, *n* = 250) from the Limited set who were not reclassified to 1 higher nodal stage to minimize data heterogeneity, as less extensive surgeries (i.e., D1 or D1 + gastrectomy) were performed in these patients, compared to the rest of the dataset, and additionally there was no significant difference in OS between the pT1N0M0 of the Limited and Adequate set (*P* = 0.466; data not shown).—no cases, *rLNs* retrieved lymph nodes, *OS* overall survival rate, *AJCC* American Joint Committee on Cancer, *pN* pathological nodal classification, *pN’* modified pathological nodal classification, *pTNM* pathological tumor-node-metastasis classification, *pTN’M* pathological tumor-modified node-metastasis classification

**Table 2:** Analysis of the 5-year overall survival rates of patients using the 8th AJCC pN classification stratified into limited (< 16 rLNs) and adequate (≥ 16 rLNs) rLN set (SEER multiethnicity dataset)

Stage	Limited rLN dataset		Stage	Adequate rLN dataset		*P* value (Limited vs. Adequate rLN dataset)	Stage	Combined rLN dataset	
	*n* (%)	5-year OS (%)		*n* (%)	5-year OS (%)			*n* (%)	5-year OS (%)
pN (*n* = 5429)			pN (*n* = 4779)				pN (*n* = 10,208)		
N0	2516 (46.3)	57.3		1499 (31.4)	71.5	< 0.001		4015 (39.3)	62.2
N1	1258 (23.2)	32.1		810 (16.9)	54.7	< 0.001		2068 (20.3)	40.3
N2	1037 (19.1)	24.3		813 (17.0)	37.3	< 0.001		1850 (18.1)	29.9
N3a	618 (11.4)	10.8		1036 (21.7)	22.0	< 0.001		1654 (16.2)	17.8
N3b	–	–		621 (13.0)	9.0	–		621 (6.1)	9.0
^*^pN’ (*n* = 4325)			^*^pN’ (*n* = 5883)				pN’ (*n* = 10,208)		
N’0	–	–		2603 (44.2)^*^	71.8	–		2603 (25.5)	71.8
N’1	1412 (32.6)	46.4		810 (13.8)	54.7	0.011		2222 (21.8)	49.1
N’2	1258 (29.1)	32.1		813 (13.8)	37.3	0.001		2071 (20.3)	34.1
N’3a	1037 (24.0)	24.3		1036 (17.6)	22.0	0.854		2073 (20.3)	23.2
N’3b	618 (14.3)	10.8		621 (10.6)	9.0	0.548		1239 (12.1)	9.9
pTNM (*n* = 5429)			pTNM (*n* = 4779)				pTNM (*n* = 10,208)		
IA	1104 (20.3)			614 (12.8)	85.2	< 0.001		1718 (16.8)	76.6
IB	652 (12.0)	56.4		396 (8.3)	74.2	< 0.001		1048 (10.3)	62.6
IIA	876 (16.1)	44.1		631 (13.2)	62.7	< 0.001		1507 (14.8)	51.3
IIB	878 (16.2)	32.9		629 (13.2)	46.9	< 0.001		1507 (14.8)	38.2
IIIA	1164 (21.4)	22.0		848 (17.7)	38.9	< 0.001		2012 (19.7)	28.9
IIIB	672 (12.4)	12.4		971 (20.3)	22.1	< 0.001		1643 (16.1)	18.1
IIIC	83 (1.5)	1.9		690 (14.4)	8.7	0.093		773 (7.6)	7.8
^*^pTN’M (*n* = 4325)			^*^pTN’M (*n* = 5883)				pTN’M (*n* = 10,208)		
IA’				1718 (29.2)^*^	76.6			1718 (16.8)	76.6
IB’				396 (6.7)	74.2			396 (3.9)	74.2
IIA’	652 (15.1)	56.4		631 (10.7)	62.7	0.066		1283 (12.6)	59.3
IIB’	876 (20.3)	44.1		629 (10.7)	46.9	0.356		1505 (14.7)	45.1
IIIA’	1116 (25.8)	30.4		848 (14.4)	38.9	< 0.001		1964 (19.2)	33.9
IIIB’	1009 (23.3)	21.5		971 (16.5)	22.1	0.071		1980 (19.4)	21.8
IIIC’	672 (15.5)	10.7		690 (11.7)	8.7	0.704		1362 (13.3)	9.7

^*^Contained patients with pT1N0M0 lesions (SEER dataset, *n* = 1104) from the Limited set who were not reclassified to 1 higher nodal stage to minimize data heterogeneity, as less extensive surgeries (i.e., D1 or D1 + gastrectomy) were performed in these patients, compared to the rest of the dataset, and additionally there was no significant difference in OS between the pT1N0M0 of the Limited and Adequate set (*P* = 0.466; data not shown).—no cases, *rLNs* pathologically retrieved lymph nodes, *OS* overall survival rate, *AJCC* American Joint Committee on Cancer, *pN* pathological nodal classification, *pN’* modified pathological nodal classification, *pTNM* pathological tumor-node-metastasis classification, *pTN’M* pathological tumor-modified node-metastasis classification

Based on these observations, we proposed a nodal approach in which only the nodal subgroups N0, N1, N2 and N3a from the Limited set were re-classified to one higher nodal subgroup, equivalent to N1, N2 N3a and N3b, respectively, while those of the Adequate set remained unchanged. Of note, patients with pT1N0M0 lesions from the Limited set were not reclassified in order to minimize data heterogeneity, as less extensive surgeries (i.e., D1 or D1 + gastrectomy) were performed in these patients, compared to the rest of the dataset, and additionally, there was no significant difference in OS between the pT1N0M0 of the Limited and Adequate set (stage IA, *P* = 0.466; Tables 1 and 2). This new nodal classification (N’) was labeled as N’0 (consisting of N0_adequate_ but also included pT1N0M0 cases of the Limited set), N’1 [consisting of both N1_adequate_ and N0_limited_ (excluding pT1N0M0 cases of the Limited set)], N’2 (N2_adequate_ and N1_limited_), N’3a (N3a_adequate_ and N2_limited_), and N’3b (N3b_adequate_ and N3a_limited_), respectively (Fig. 1a, b).

### Significance of the N’ approach in the Chinese dataset

The resulting OS curves demonstrated a more favorable homogenization of survival rates between the newly classified two sets of patients, as illustrated by the alignment of their OS curves in Fig. 1b, except for the N’2 subgroup (*P* = 0.018). Further, less stage migration and considerable improvement in OS could be observed in the Combined rLN N’ dataset than the Combined 8th AJCC pN dataset (Tables 1, 2); i.e., the OS of N’0 to N’3a patients were less underestimated and that of N’3b were less overestimated.

Next, we replaced the pN classification of the 8th AJCC pTNM staging system with our N’ classification to form the pTN’M classification. Stratified analyses demonstrated an improved statistical association between the Limited and Adequate set in which all *P* values between the two sets from the same sub-stage were greater than 0.05; representing an improved homogeneity of the proposed pTN’M classification (IIA’: *P* = 0.657; IIB’: *P* = 0.826; IIIA’: *P* = 0.753; IIIB’: *P* = 0.949; IIIC’: *P* = 0.706) (Fig. 2b). The detailed change in OS from the 8th AJCC pTNM classification to the pTN’M classification is shown in Tables 1, 2.

Furthermore, to avoid interference of collinearity between the 8th AJCC pTNM and the pTN’M classification, two separate multivariate analyses were performed. The clinicopathological factors found to be independently correlated with OS were age, tumor location, tumor size, Lauren type, pT, pN and N’ classification. Importantly, the number of LNs retrieved (Limited vs. Adequate) was only found to be an independent factor for the 8th AJCC pTNM classification (*P* < 0.001), whereas it was not found to be independent for our proposed pTN’M classification (*P* = 0.940; Table 3).

**Table 3:** Multivariate analysis of clinicopathological characteristics for the Chinese dataset

Model 1^*^				Model 2^†^			
Characteristics	*HR*	95% CI	*P *value	Characteristics	*HR*	95% CI	*P *value
Age (years)							
≤ 60	Ref				Ref		
> 60	1.344	1.254–1.440	< 0.001		1.342	1.252–1.437	< 0.001
Tumor size (cm)							
≤ 4.5	Ref				Ref		
> 4.5	1.252	1.164–1.347	< 0.001		1.254	1.166–1.348	< 0.001
Tumor location							
Lower 1/3	Ref				Ref		
Middle 1/3	1.216	1.106–1.336	< 0.001		1.214	1.105–1.335	< 0.001
Upper 1/3	1.132	1.039–1.234	0.005		1.126	1.034–1.227	0.006
Entire stomach	1.575	1.404–1.767	< 0.001		1.561	1.392–1.750	< 0.001
Lauren type							
Intestinal	Ref				Ref		
Diffuse	1.162	1.081–1.249	< 0.001		1.160	1.079–1.247	< 0.001
Total LNs retrieval							
≥ 16	Ref				Ref		
< 16	1.583	1.463–1.712	< 0.001		0.997	0.924–1.075	0.940
pT							
T1	Ref				Ref		
T2	4.329	3.225–5.810	< 0.001		3.785	2.807–5.104	< 0.001
T3	4.943	3.676–6.645	< 0.001		4.337	3.210–5.860	< 0.001
T4a	6.096	4.568–8.136	< 0.001		5.345	3.985–7.171	< 0.001
T4b	8.144	5.994–11.066	< 0.001		7.143	5.234–9.749	< 0.001
pN				N’			
N0	Ref			N0	Ref		
N1	1.853	1.645–2.087	< 0.001	N1	1.738	1.493–2.024	< 0.001
N2	2.530	2.264–2.827	< 0.001	N2	2.752	2.383–3.183	< 0.001
N3a	4.325	3.865–4.839	< 0.001	N3a	4.264	3.706–4.907	< 0.001
N3b	6.376	5.586–7.278	< 0.001	N3b	6.573	5.673–7.616	< 0.001

^*^Multivariate analysis Model 1: Clinicopathological factors including the 8th AJCC N classification. ^†^Multivariate analysis Model 2: Clinicopathological factors including our proposed N’ classification. *HR* hazard ratio, *CI* confidence interval, *Ref* the corresponding variable subgroup which was used as reference when performing the multivariate analysis, *T* tumor size, *LN* lymph node, *pT or pN* pathological tumor or nodal classification, *N’* modified nodal classification, *NS* non-significant, *AJCC* American Joint Commission on Cancer

### Validation of the modified nodal classification and staging system in the SEER dataset

When the same analyses were performed using the SEER dataset, similar findings as to that of the Chinese dataset were observed. Briefly, significant differences between the OS of patients in the Limited and Adequate set were observed when staged according to the AJCC pN classification (N0–N3a, *P* < 0.001; Fig. 1c) and the 8th AJCC pTNM staging system (IA–IIIB, *P* < 0.001), except for stage IIIC patients’ (*P* = 0.093) (Fig. 2c). Applying this modified nodal system to the SEER dataset also demonstrated better homogeneity between the OS of these two sets of patients, either in terms of statistical difference or shorter separation between the Limited and Adequate set survival curves (Limited vs. Adequate set, N’1, *P* = 0.011; N’2, *P* = 0.001; N’3a, *P* = 0.854; N’3b, *P* = 0.548; Fig. 1d; IIA’, *P* = 0.066; IIB’, *P* = 0.356; IIIA’, *P* < 0.001; IIIB’, *P* = 0.071; IIIC’, *P* = 0.704; Fig. 2d). Further, the detailed amelioration in OS from the 8th AJCC pN classification to the N’ classification and the 8th AJCC pTNM staging system to the pTN’M system can be observed in Tables 1 and 2.

### Performance of the modified nodal classification and staging system

The clinical reliability for differentiating between each subgroup within the nodal classification and TNM stages when patients from both the Adequate and Limited sets were analyzed as a single dataset was measured in terms of the log-rank *χ*^2^, linear trend *χ*^2^ and likelihood ratio *χ*^2^. Their overall prognostic abilities were analyzed by calculating their AIC value. As shown in Table 4, the performance of the pTN’M staging system for both the Chinese and SEER datasets was found to be superior to the 8th AJCC pTNM staging system; demonstrating promising applicability of the N’ classification in datasets of GC patients comprising of both Limited and Adequate number of rLNs, irrespective of patient ethnicity and possible differences in treatment strategies between the Chinese and non-Chinese datasets.

**Table 4:** Performance of the 8th AJCC TNM classification to the proposed pTN’M classification in the Chinese and SEER datasets

Statistical parameters	Chinese dataset		SEER dataset	
	8th AJCC pTNM classification	Proposed pTN’M classification	8th AJCC pTNM classification	Proposed pTN’M classification
AIC	55,324	55,207	84,730	84,568
Log-rank *χ*^2^	2139	2298	1926	2122
Linear trend *χ*^2^	1531	1630	1649	1798
Likelihood ratio *χ*^2^	2135	2252	1863	2025

*AIC* Akaike information criterion, *pTNM* pathological tumor-node-metastasis classification, *N’* modified nodal classification, *AJCC* American Joint Commission on Cancer

## Discussion

Adequate perioperative LN retrieval is critical for proper pathological staging, the most accurate diagnosis for the patients, as it guides the necessity for adjuvant therapy, the use of single or double regimen, or requirement of combination therapy (i.e., chemoradiotherapy); which directly affects patients’ outcomes. It is relevant for other GI cancers as well such as colorectal cancer (recommended rLN = 12) which also emphasizes the removal of adequate LNs for proper post-operative staging. However, LN harvesting is laborious and time-consuming. In both gastric and colorectal cancers, numerous researches have been performed in an attempt to resolve the unmet clinical need of adequate LN retrieval and proper classification of those with limited rLNs. Operatively, different types of dyes [17, 18] and techniques [19, 20] have been proposed, and post-operatively, complicated calculations such as extranodal extension (ENE), log odds of positive lymph nodes (LODDS), lymph node ratio (LNR), and more have been proposed, however, the rate of wide clinical application of these techniques have been low. This could be due to the requirement of additional perioperative labor by the surgeons and pathologists, or complicated calculations which deviate extensively from the conventionally used AJCC staging system. Thus, in this study, we propose and validate a novel nodal staging system using the AJCC N classification as base, to confront the major challenge regarding stage migration existing between patients having Limited and Adequate rLNs when they are staged within the same TNM staging system.

Our proposed classification approach markedly minimized the survival difference between patients of the Limited and Adequate sets and demonstrated better prognostic ability (AIC of the pTN’M vs. 8th AJCC pTNM: Chinese dataset, 55,207 vs. 55,324, and SEER dataset, 84,568 vs. 84,730) than the 8th AJCC pTNM staging system, in both a Chinese multicenter and SEER multi-ethnicity datasets.

There were some notable exceptions observed despite implementing the N’ classification in certain nodal subgroups of the Chinese and SEER datasets. For instance, significant statistical differences were observed between the Limited and Adequate set for N’2 (*P* = 0.018; Fig. 1b) in the Chinese dataset, and N’1 and N’2 (*P* = 0.011 and *P* = 0.001; Fig. 1d) in the SEER dataset. This could be a multifactorial issue related to the number of patients in these subgroups, total number of rLNs, ratio of metastatic LNs between the Limited and Adequate set, etc. Additional analysis of the datasets to further address this issue was performed by assessing the independent clinicopathological factors associated with OS in both datasets (data not shown). For the Chinese dataset, we suggest that this could be due to the large number of T4a patients in the N1 subgroup of the Limited set (N0 vs. N1 vs. N2 vs. N3a: 40.9% vs. 58.7% vs. 61.1% vs. 62.9%), which was almost similar to that of the N2 and N3a subgroups, thereby leading to a significantly lower than expected OS in this patients’ subgroup. For the SEER dataset, we hypothesized that this could be associated with the relatively high number of T3 patients in the N’1 subgroup and relatively low number of rLNs in the N’2 subgroup. However, its impact on the TNM classification was not as significant (Fig. 2), except for the SEER dataset stage IIIA’. Additionally, compared to the 8th AJCC pN subgroups for which all *P* value were initially less than 0.001 and upon reclassification using the modified nodal groupings, narrower survival gaps between these corresponding Limited and Adequate nodal subgroups were observed, thereby proving that the proposed classification strategy could bring more homogeneity in terms of OS between patients of the Limited and Adequate set of the same subgroups.

Previously, the LNR and LODDS were among the common attempts to tackle this stage migration effect [21–24]. Comparatively, the proposed N’ classification includes the following advantages: first, the N’ classification has considerable resemblance to the 8th AJCC LN classification methodology, with the exception that patients with < 16 LNs were promoted to one higher nodal class, and would be easier to implement. Second, the N’ classification does not require mind-straining mathematical calculations, unlike the LNR or LODD. Third, N’ classification is more stable to use as the cut-off values of the LNR and LODDs tend to vary between different institutions and even different patient datasets of the same institution; making them less reliable as a standardized approach.

The strengths of the N’ classification are as follows. First, it was developed based on the data of 358,781 surgically retrieved LNs derived from a large cohort of high-volume Chinese GC institutions and validated in a larger Western population. This demonstrates that although there exist differences in patient characteristics, treatment strategies, and survival outcomes between the Chinese and SEER datasets, however, the proposed classification could still be applied in different populations of GC and was superior to the 8th AJCC classification in both populations (Additional file 2: Table S1); indicating reliable clinical applicability. Second, it homogenized the classification of the Limited set of patients by classifying them in sub-groups corresponding closer to their actuarial OS; thereby decreasing the effect of stage migration (Figs. 1, 2, and Tables 1 and 2). Consequently, this led to a more homogeneous distribution of patients with < 16 and ≥ 16 rLNs between each subgroup (N’0–N’3b) of the N’ classification, as compared to the 8th AJCC pN classification, and rendered LNs retrieved for the multivariate Model 2 a non-independent factor for OS (*P* = 0.940, as NS in Table 4). Lastly, as N’ classification has a close resemblance to the nodal classification methodology of the 8th AJCC N classification, it could be clinically more efficiently and widely applied, i.e., in institutions where high-quality gastrectomies are difficult to be performed (i.e., leading to a high number of gastrectomies with < 16 rLNs) or in under-resourced countries where important prognostic factors usually utilized in predictive models such as nomograms [25, 26] might be unavailable (i.e., CA-724, HER-2, genetic data) to provide more accurate individualized OS predictions.

Despite the potential clinical significance of this study, there were certain shortcomings worth mentioning. First, due to the retrospective nature of this multicenter study, there may have been a lack of standardized methods in the handling of pathological specimens for which some differences between the number of LNs resected surgically compared to those manually retrieved post-operatively for pathological investigation may exist between the two populations. The former reflects the surgical quality of gastrectomies, while the latter affects the staging quality and subsequent prognostic classification of patients. Second, due to the notable exceptions observed in certain nodal subgroups, despite implementing the N’ classification strategy, larger studies in other populations are still required to further validate these findings.

## Conclusion

In conclusion, this study proposes a simple and practical method for a more standardized approach to homogenously classify cohorts of GC patients having Limited and Adequate number of retrieved LNs. The proposed nodal classification analogy imposes minor modification to the routinely used AJCC nodal classification and has demonstrated compelling results upon external validation in a large cohort of Western population; thus, suggesting possible wide clinical applicability in different GC populations.

## Supplementary Information

**Additional file 1: Fig. S1**. Flow chart illustrating the case selection process of the Chinese dataset.

**Additional file 2: Table S1**. Univariate analysis of the clinicopathological characteristics for patients from the Chinese dataset and SEER dataset.

## Data Availability

The datasets used and/or analyzed during the current study are available from the corresponding author upon reasonable request. The SEER datasets used in this study can be freely available at the following address: https://seer.cancer.gov/.

## Abbreviations

AIC: Akaike information criterion
AJCC: American Joint Committee on Cancer
CI: Confidence intervals
GC: Gastric cancer
HR: Hazard ratios
LN: Lymph node
LNR: Lymph node ratio
LODDS: Log odds of metastatic lymph nodes
rLNs: Retrieved lymph nodes
N’: Modified nodal classification
N0_adequate_: Patients with adequate number of surgically retrieved lymph nodes (≥ 16) staged as N0
N0_limited_: Patients with limited number of surgically retrieved lymph nodes (< 16) staged as N0
NCCN: National Comprehensive Cancer Network
OS: Overall survival
pN: Pathological nodal classification
pN’: Modified pathological nodal classification
pTNM: Pathological tumor-node-metastasis classification
pTN’M: Pathological tumor-modified node-metastasis classification
R0: Tumor resection with no residual macroscopic or microscopic cancer lesion
SEER: Surveillance, epidemiology, and end results
SPSS: Statistical Package for the Social Sciences
SD: Standard deviation
TNM: Tumor-node-metastasis
