# Supplementary material for: Standardizing the classification of gastric cancer patients with limited and adequate number of retrieved lymph nodes: an externally validated approach using real-world data
Source: Mil Med Res. 2022 Apr 7;9:15. doi: 10.1186/s40779-022-00375-2 (PMC8988371; doi:10.1186/s40779-022-00375-2)
Supplement: Supplementary file 1 — Additional file 1: Fig. S1. Flow chart illustrating the case selection process of the Chinese dataset. [file 40779_2022_375_MOESM1_ESM.pdf]

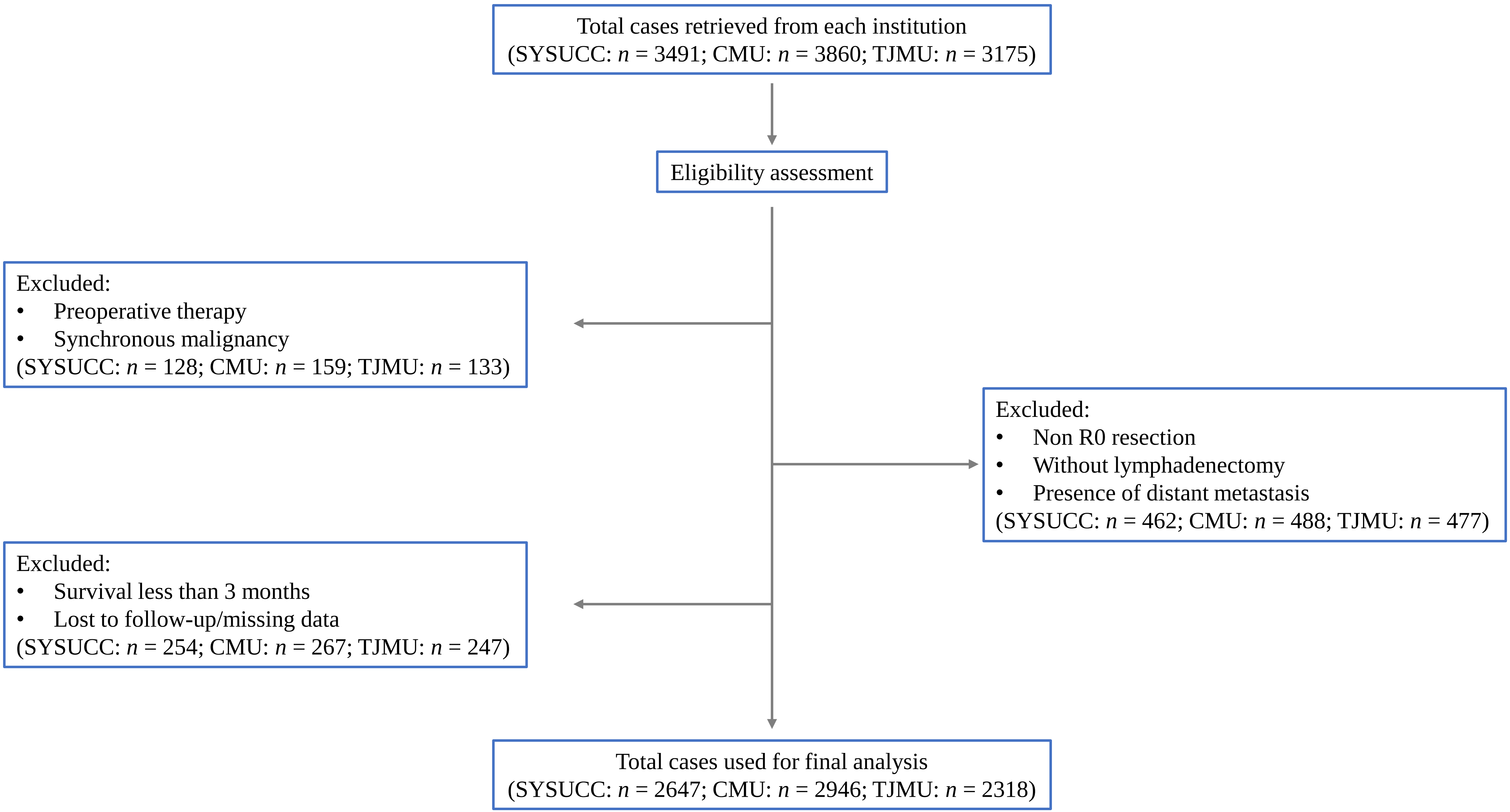
Total cases retrieved from each institution
(SYSUCC: n = 3491; CMU: n = 3860; TJMU: n = 3175)
Eligibility assessment
Excluded:
• Preoperative therapy
• Synchronous malignancy
(SYSUCC: n = 128; CMU: n = 159; TJMU: n = 133)
Excluded:
• Non R0 resection
• Without lymphadenectomy
• Presence of distant metastasis
(SYSUCC: n = 462; CMU: n = 488; TJMU: n = 477)
Excluded:
• Survival less than 3 months
• Lost to follow-up/missing data
(SYSUCC: n = 254; CMU: n = 267; TJMU: n = 247)
Total cases used for final analysis
(SYSUCC: n = 2647; CMU: n = 2946; TJMU: n = 2318)
