# Supplementary material for: Standardizing the classification of gastric cancer patients with limited and adequate number of retrieved lymph nodes: an externally validated approach using real-world data
Source: Mil Med Res. 2022 Apr 7;9:15. doi: 10.1186/s40779-022-00375-2 (PMC8988371; doi:10.1186/s40779-022-00375-2)
Supplement: Supplementary file 2 — Additional file 2: Table S1. Univariate analysis of the clinicopathological characteristics for patients from the Chinese dataset and SEER dataset. [file 40779_2022_375_MOESM2_ESM.pdf]

Table S1 Univariate analysis of the clinicopathological characteristics for patients from the Chinese dataset and SEER dataset

Parameters		Chinese dataset (n = 7911)				SEER dataset (n = 10,208)			
		n	***P*-value**	***HR***	**95%CI**	***n***	***P*-value**	***HR***	**95%CI**
Sex	Female	2325				3848			
	Male	5586	0.082	1.069	0.992 - 1.151	6360	0.049	1.059	1.000 - 1.122
Age	< 60	4117				3046			
	≥ 60	3794	< 0.001	1.372	1.283 - 1.468	7162	< 0.001	1.460	1.369 - 1.557
Tumor location	Lower 1/3	3578				3382			
	Middle 1/3	1523	< 0.001	1.432	1.304 - 1.573	2701	0.006	0.900	0.834 - 0.970
	Upper 1/3	2144	< 0.001	1.490	1.372 - 1.617	3694	< 0.001	1.141	1.068 - 1.219
	Entire stomach	666	< 0.001	2.635	2.357 - 2.947	431	< 0.001	2.065	1.827 - 2.333
Tumor size (cm)	≤ 4.5	4081				5722			
	> 4.5	3830	< 0.001	2.143	1.999 - 2.297	4486	< 0.001	1.681	1.590 - 1.777
Lauren type	Intestinal	3329				4062			
	Diffuse	4582	< 0.001	1.397	1.302 - 1.498	6146	< 0.001	1.372	1.295 - 1.454
8th AJCC	T1	954				2172			
pT	T2	1447	< 0.001	7.258	5.426 - 9.710	1402	< 0.001	1.693	1.496 - 1.916
	T3	1291	< 0.001	10.641	7.963 - 14.222	3901	< 0.001	3.143	2.850 - 3.466
	T4a	3675	< 0.001	14.954	11.292 - 19.804	2061	< 0.001	4.195	3.784 - 4.650
	T4b	544	< 0.001	23.274	17.296 - 31.319	672	< 0.001	5.496	4.851 - 6.228
8th AJCC	N0	2870				4015			
pN	N1	1403	< 0.001	2.435	2.165 - 2.739	2068	< 0.001	1.897	1.746 - 2.061
	N2	1547	< 0.001	3.535	3.171 - 3.940	1850	< 0.001	2.518	2.321 - 2.731
	N3a	1407	< 0.001	5.703	5.129 - 6.341	1654	< 0.001	3.545	3.272 - 3.841
	N3b	684	< 0.001	8.406	7.451 - 9.484	621	< 0.001	4.761	4.280 - 5.296
N’	N’0	2057				2603			
	N’1	1698	< 0.001	2.704	2.341 - 3.122	2222	< 0.001	2.023	1.826 - 2.242
	N’2	1507	< 0.001	4.440	3.867 - 5.097	2071	< 0.001	3.025	2.739 - 3.341
	N’3a	1690	< 0.001	7.352	6.443 - 8.389	2073	< 0.001	4.259	3.868 - 4.690
	N’3b	959	< 0.001	12.377	10.777 - 14.216	1239	< 0.001	6.516	5.877 - 7.224
Total LNs retrieved	≥ 16	5497				4779			
	< 16	2414	< 0.001	1.162	1.082 - 1.247	5429	< 0.001	1.099	1.040 - 1.162

8th AJCC	IA	801				1718			
pTNM	IB	735	< 0.001	4.071	2.756 - 6.012	1048	< 0.001	1.689	1.451 - 1.967
	IIA	699	< 0.001	6.638	4.549 - 9.688	1507	< 0.001	2.252	1.969 - 2.576
	IIB	1499	< 0.001	10.603	7.418 - 15.155	1507	< 0.001	3.253	2.859 - 3.702
	IIIA	2076	< 0.001	18.715	13.165 - 26.604	2012	< 0.001	4.458	3.949 - 5.033
	IIIB	1340	< 0.001	30.356	21.326 - 43.210	1643	< 0.001	6.045	5.351 - 6.830
	IIIC	761	< 0.001	45.728	32.013 - 65.319	773	< 0.001	8.433	7.372 - 9.648
pTN'M	IA’	801				1718			
	IB’	508	< 0.001	3.258	2.136 - 4.969	396	0.056	1.252	0.994 - 1.577
	IIA’	692	< 0.001	5.561	3.793 - 8.152	1283	< 0.001	1.790	1.550 - 2.067
	IIB’	1146	< 0.001	9.084	6.323 - 13.049	1505	< 0.001	2.638	2.312 - 3.010
	IIIA’	2181	< 0.001	15.512	10.908 - 22.060	1964	< 0.001	3.768	3.332 - 4.260
	IIIB’	1561	< 0.001	27.643	19.434 - 39.318	1980	< 0.001	5.383	4.774 - 6.070
	IIIC’	1022	< 0.001	45.446	31.894 - 64.755	1362	< 0.001	8.159	7.208 - 9.235

AJCC American Joint Commission on Cancer, *T* tumor size, *LN* lymph node, *pT or pN* pathological tumor or nodal classification, *N’* modified nodal classification, *pTNM* pathological tumor-node-metastasis classification, *pTN’M* pathological tumor-modified node-metastasis classification
